# Potential Causal Relationship Between Extensive Lipid Profiles and Various Hair Loss Diseases: Evidence From Univariable and Multivariable Mendelian Randomization Analyses

**DOI:** 10.1111/jocd.70176

**Published:** 2025-04-10

**Authors:** Yuhan Xie, Ruimin Bai, Landong Ren, Hengtong Fan, Huihui Tuo, Longmei Duan, Xiaolin Zhou, Chengyu Fang, Ziyan Li, Yan Zheng

**Affiliations:** ^1^ Department of Dermatology The First Affiliated Hospital of Xi'an Jiaotong University Xi'an Shaanxi China; ^2^ Department of Urology The First Affiliated Hospital of Xi'an Jiaotong University Xi'an Shaanxi China

**Keywords:** alopecia areata, androgenetic alopecia, cicatricial alopecia, hair loss diseases, lipid, Mendelian randomization

## Abstract

**Background:**

Hair loss disorders, including non‐cicatricial forms such as alopecia areata (AA) and androgenetic alopecia (AGA), as well as cicatricial forms, represent significant dermatological concerns influenced by various factors, including lipid metabolism. While observational studies and clinical trials have suggested a link between lipid levels and hair loss, the causal relationship remains unclear.

**Methods:**

We conducted a comprehensive analysis of 983 lipid variables [including triglycerides (TG), fatty acids, cholesterol, cholesterol esters, phospholipids, and lipoproteins] and 4 hair loss disorders. Two‐sample univariable Mendelian randomization (UVMR) and multivariable Mendelian randomization (MVMR) analyses were employed to investigate the causal effects of lipids on hair loss disorders. Sensitivity analyses were performed to ensure the robustness of our findings.

**Results:**

The UVMR analysis identified 56 significant causal associations between lipid levels and hair loss disorders, with cholesterol, high‐density lipoprotein cholesterol (HDL‐C), low‐density lipoprotein cholesterol (LDL‐C), TG, apolipoprotein A1, apolipoprotein B, and lipoprotein(a) emerging as key contributors. The MVMR analysis evaluated the independent effects of HDL‐C, LDL‐C, and TG on alopecia disorders, identifying significant associations only between HDL‐C, TG, and AA. Sensitivity analyses confirmed the consistency and robustness of these results.

**Conclusion:**

This study provides strong evidence for potential causal associations between lipids and hair loss disorders, highlighting potential therapeutic targets and the importance of lipid management in affected patients.

## 1 Introduction

Hair serves essential functions in both physiological and psychological domains of life. Hair loss, or alopecia, is a common chronic dermatological condition affecting millions globally, profoundly impacting their quality of life and psychological well‐being [1]. The etiology of hair loss is multifactorial, involving genetic and epigenetic changes, immune dysregulation, endocrine disorders, chronic illnesses, nutritional deficiencies, stress, medications, and chemotherapy [2]. Alopecia is primarily classified into two categories: non‐scarring (non‐cicatricial) alopecia and scarring (cicatricial) alopecia. Non‐scarring alopecia is characterized by hair loss due to alterations in the hair cycle, follicle size, or hair shaft integrity, while the hair follicle remains intact. The most common forms of non‐scarring alopecia are alopecia areata (AA) and androgenetic alopecia (AGA). AA is an autoimmune disorder marked by transient hair loss, typically presenting as sudden, well‐defined patches on the scalp. The global prevalence of AA is estimated at approximately 0.2%, with 1.7%–2.1% of individuals experiencing AA during their lifetime [2]. Although the precise etiology and pathogenesis of AA are not fully understood, the disruption of immune privilege in hair follicles is considered a key factor in its development [3, 4]. AGA, also known as male pattern hair loss (MPHL) or female pattern hair loss (FPHL), is characterized by progressive terminal hair loss post‐puberty, primarily affecting the frontal, temporal, and vertex regions of the scalp. AGA is the most common form of hair loss worldwide, affecting up to 80% of men and 50% of women by the age of 70, with its prevalence increasing with age. The condition is primarily driven by genetically determined follicular sensitivity to the androgenic metabolite dihydrotestosterone (DHT) [5]. Scarring alopecia, although less common, is distinguished by follicular destruction, skin scarring, and irreversible hair loss [4].

Lipids can be broadly categorized into fats, lipoids, and lipoproteins, each with distinct biological functions. Fats, such as triglycerides (TG) and fatty acids, primarily serve as energy storage molecules. Lipoids, including cholesterol, cholesterol esters, and phospholipids, contribute to membrane integrity and intracellular signaling, while lipoproteins, such as high‐density lipoprotein cholesterol (HDL‐C) and low‐density lipoprotein cholesterol (LDL‐C), facilitate lipid transport in the bloodstream [6]. Beyond their well‐established roles in metabolic syndrome (MetS), cardiovascular diseases, and immune‐inflammatory conditions [7, 8, 9, 10, 11, 12, 13], lipids are increasingly recognized as key regulators of skin homeostasis and hair follicle biology. Emerging evidence suggests that lipid metabolism dysregulation may contribute to alopecia, with studies reporting elevated LDL‐C levels in AA patients [14] and increased TG and LDL‐C levels in AGA patients [15, 16]. Additionally, specific lipid molecules, such as cholesterol and fatty acids, have been shown to directly influence hair follicle growth and development [17, 18].

Despite substantial evidence linking lipids to hair loss disorders, most studies have been observational and are limited by small sample sizes and confounding factors, and may draw implausible conclusions, leaving the causal relationships and underlying mechanisms unclear. Mendelian randomization (MR) is a powerful genetic epidemiology approach that utilizes genetic variants as instrumental variables (IVs) to infer causal relationships between exposures and outcomes. Since genetic variants are randomly allocated at conception and remain stable throughout life, MR effectively reduces confounding bias and mitigates reverse causation, two major limitations of traditional observational studies. Unlike randomized controlled trials (RCTs), MR enables causal inference in large‐scale population studies without ethical and logistical constraints, making it a valuable tool for assessing the role of lipid metabolism in disease risk [19]. However, MR is not without limitations. Weak instrument bias and potential pleiotropy may affect causal estimates, requiring rigorous sensitivity analyses to ensure robustness.

This study employed publicly available genome‐wide association study (GWAS) datasets to investigate lipid‐related traits, including commonly studied lipids such as HDL‐C, LDL‐C, and TG and less‐explored features such as fatty acids, lipoprotein(a) [Lp(a)], apolipoprotein A1 (Apo A1), and apolipoprotein B (Apo B). Through two‐sample univariable Mendelian randomization (UVMR) and multivariable Mendelian randomization (MVMR) analyses, we examined the causal relationships between lipids and alopecia disorders, including non‐scarring alopecia (AA and AGA) and scarring alopecia, aiming to elucidate the mechanisms underlying alopecia and potentially provide a theoretical basis for new therapeutic approaches.

## 2 Method

### 2.1 Study Design

**FIGURE 1:**
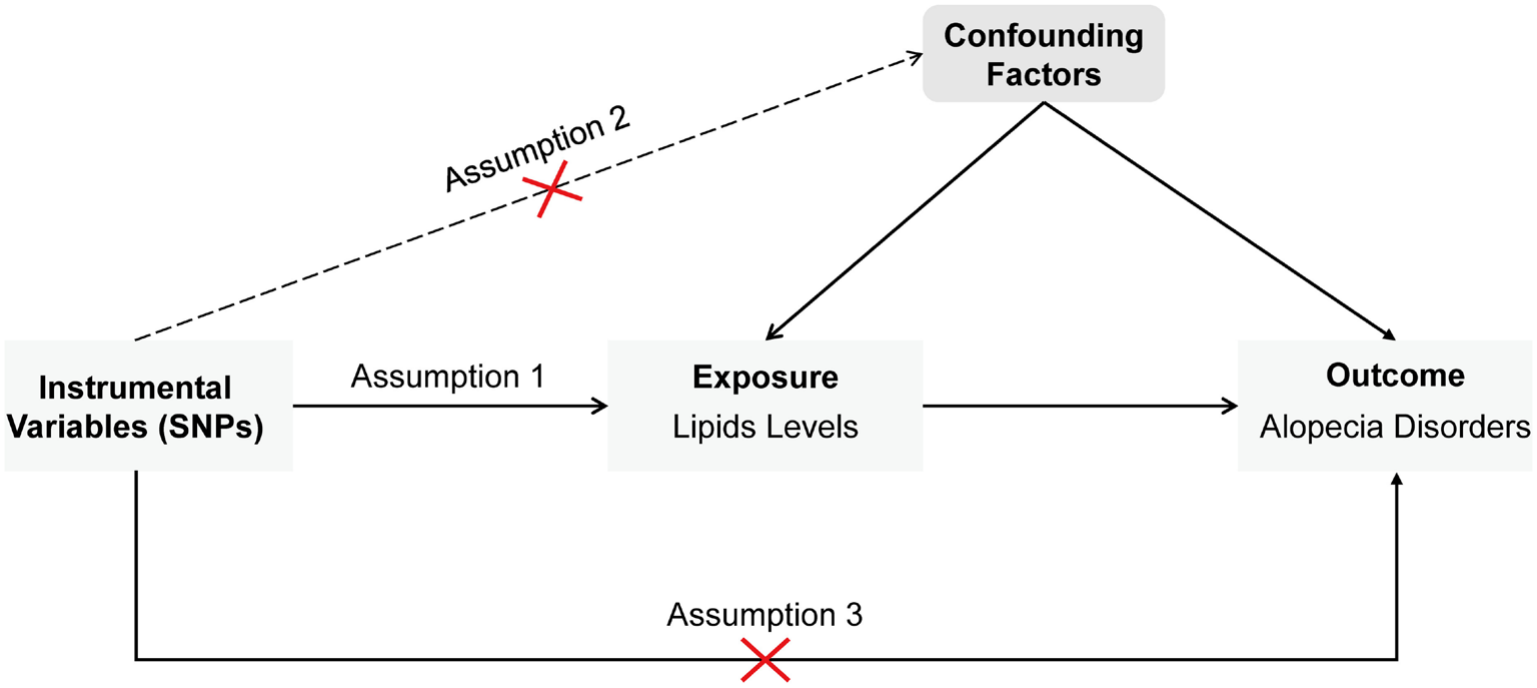
The schematic diagram of Mendelian randomization. Three assumptions should be met in univariable Mendelian Randomization analysis: Assumption 1 (Relevance Assumption): SNPs should be strongly associated with the exposure (lipid levels) to serve as valid instrumental variables; Assumption 2 (Independence Assumption): Selected SNPs should be independent of confounders that might influence both lipid levels and alopecia; Assumption 3 (Exclusion Restriction Assumption): SNPs should affect alopecia disorders only through lipid levels, without alternative biological pathways. SNP: Single nucleotide polymorphism.

**FIGURE 2:**
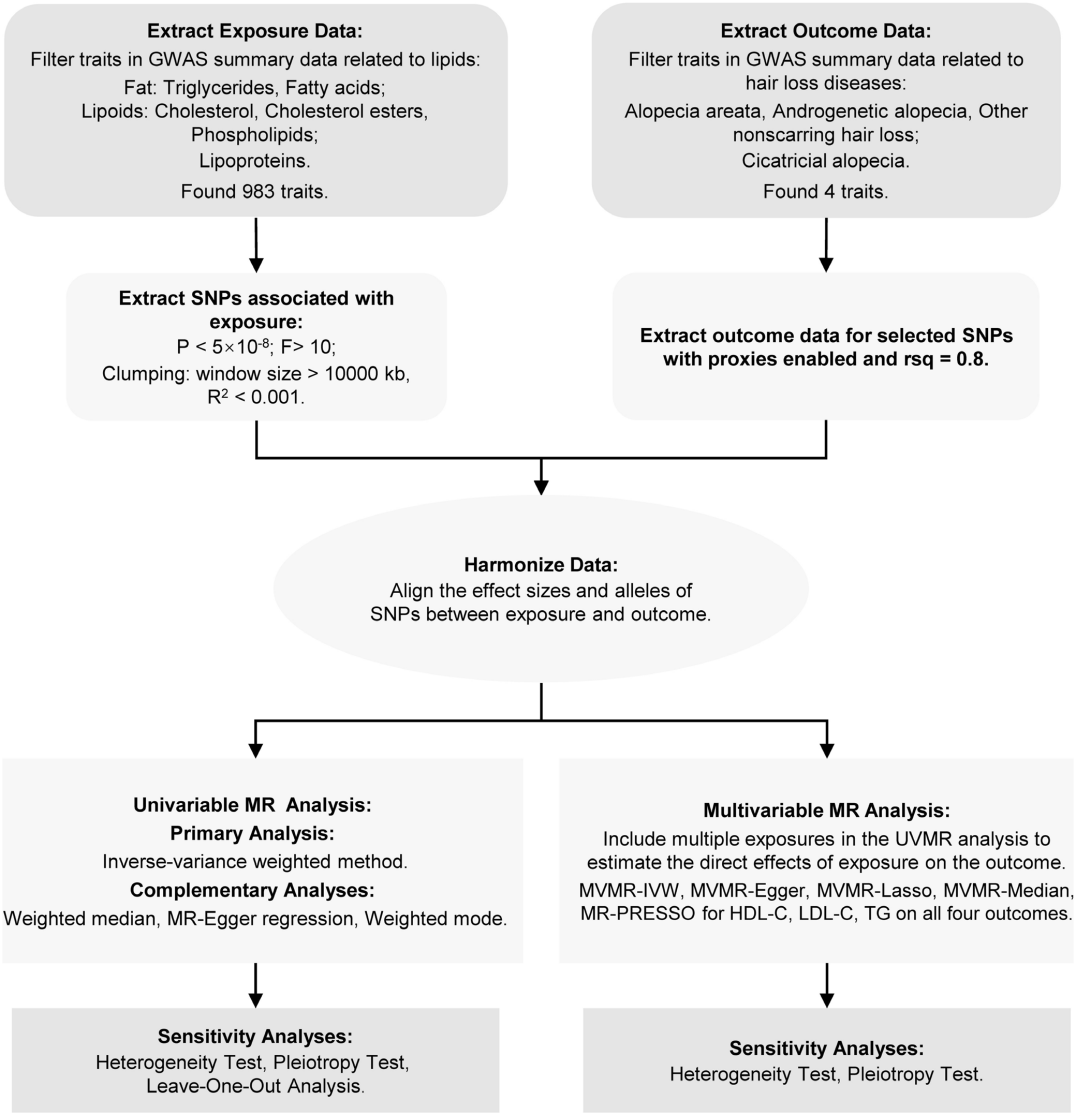
Overview of the study design. GWAS, genome‐wide association study; MR: Mendelian randomization; MVMR: Multivariable Mendelian randomization; IVW: Inverse‐variance weighted; MR‐PRESSO: Mendelian randomization pleiotropy residual sum and outlier; HDL‐C, high‐density lipoprotein cholesterol; LDL‐C, low‐density lipoprotein cholesterol; TG, triglycerides.

This study employed a two‐sample MR approach. Initially, UVMR was conducted to assess the causal relationship between lipid levels and alopecia disorders. Lipid traits were categorized into three main groups: fats (TG and fatty acids), lipoids (cholesterol, cholesterol esters, and phospholipids), and lipoproteins. Subsequently, MVMR was used to assess the independent effects of three specific lipids: HDL‐C, LDL‐C, and TG. The MR analysis must adhere to the following three fundamental assumptions (Figure 1): Assumption 1 (Relevance Assumption): Single nucleotide polymorphisms (SNPs) should be strongly associated with the exposure (lipid levels) to serve as valid IVs; Assumption 2 (Independence Assumption): Selected SNPs should be independent of confounders that might influence both lipid levels and alopecia; and Assumption 3 (Exclusion Restriction Assumption): SNPs should affect alopecia disorders only through lipid levels rather than via alternative biological pathways. The study design is depicted in Figure 2.

### 2.2 Data Sources

The lipid‐related exposure data were obtained from publicly available GWAS summary datasets from various consortia (https://gwas.mrcieu.ac.uk/). The primary GWAS statistics were sourced from Kettunen et al.'s 2016 study, which quantified 123 blood metabolite traits in up to 24 925 individuals [20], and Richardson et al.'s 2022 study, which systematically estimated the effects of genetic variants on 249 metabolic traits in blood samples from up to 115 082 UK Biobank participants [21]. Alopecia disorders, classified into non‐scarring and scarring alopecia, served as the outcome variables. Non‐scarring alopecia was further subdivided into AA, AGA, and other forms of non‐scarring hair loss. Outcome data for alopecia were derived from the 2021 FinnGen study (https://r5.finngen.fi/), where diagnoses were assigned based on International Classification of Diseases (ICD) codes. Specifically, AA (finn‐b‐L12_ALOPECAREATA) was defined using ICD‐10: L63, ICD‐9: 7040B, 7040C, and ICD‐8: 70400; AGA (finn‐b‐L12_ALOPECANDRO) was defined using ICD‐10: L64 and ICD‐9: 7040A; other non‐scarring hair loss (finn‐b‐L12_HAIRLOSSNONSCAR) was defined using ICD‐10: L65; and cicatricial alopecia (scarring hair loss) (finn‐b‐L12_HAIRLOSSSCAR) was defined using ICD‐10: L66. We utilized publicly available GWAS datasets, for which informed consent and ethical approvals had already been obtained, thus no additional ethical approval was required for this study. All MR analyses adhered to the STROBE‐MR guidelines [22].

### 2.3 Instrumental Variables Selection

In both univariable and multivariable MR analyses, SNPs closely associated with exposure at a genome‐wide significance level (*p* < 5 × 10^−8^) were first extracted from the GWAS summary‐level dataset to serve as IVs. To ensure instrument strength and minimize weak instrument bias, SNPs with F‐statistics > 10 were selected. Linkage disequilibrium was addressed by applying a clumping window of 10 000 kb and an *r* ^2^ threshold of < 0.001. Additionally, proxy SNPs with *r* ^2^ > 0.8 were selected. Allele inconsistencies among SNPs were adjusted, and SNPs with palindromic sequences or incompatible sequences were removed.

### 2.4 Statistical Analysis

In UVMR, multiple methods were utilized, including the inverse‐variance weighted (IVW) method, MR‐Egger regression, weighted median (WM), and weighted mode. The IVW estimate is the inverse variance weighted mean of ratio estimates from 2 or more instruments. This method assumes that all SNPs are valid instruments or are invalid in such a way that the overall bias is zero [23]. MR‐Egger can detect and adjust for pleiotropy, though its estimates may be less precise [24]. The WM method provides accurate estimates assuming at least 50% of the IVs are valid [25]. The weighted mode method is sensitive to bandwidth selection for mode estimation [26]. IVW was used as the primary method due to its efficiency and robustness. MR‐Egger, WM, and weighted mode methods serve as supplementary approaches to provide reliable estimates under broader scenarios. Heterogeneity was assessed using Cochran's Q statistic (*p* < 0.05 indicating heterogeneity), with the IVW random effects model and MR‐Egger [27]. Horizontal pleiotropy was tested using the intercept from MR‐Egger analysis (*p* < 0.05 indicating pleiotropy) [24]. Leave‐one‐out tests were conducted to examine the influence of individual SNPs on causal inference [28].

Given the potential pleiotropy of some lipid traits and the extensive genetic overlap between HDL‐C, LDL‐C, and TG [29], MVMR was applied to estimate the direct causal effects of each lipid component and determine whether their impact on alopecia disorders is independent. SNP instruments for MVMR were selected based on exposure variables with positive causal associations with the outcomes or from relevant exposures identified by the GLGC (e.g., TG: ieu‐a‐302). MVMR‐IVW was used as the primary method, with MVMR‐Egger, MVMR‐Lasso, and MVMR‐Median methods as supplementary approaches to estimate causal effects. MR pleiotropy residual sum and outlier (MR‐PRESSO) was employed to detect potential outliers with horizontal pleiotropy characteristics [23]. Heterogeneity was tested using Cochran's Q statistic, and pleiotropy was assessed using MVMR‐Egger.

All analyses were performed using R statistical software (version 4.3.2), utilizing the “TwoSampleMR,” “MendelianRandomization,” and “MR‐PRESSO” packages. A *p*‐value of less than 0.05 was considered statistically significant.

## 3 Results

After screening GWAS summary data related to lipid metabolism, we identified 983 lipid‐related traits out of a total of 50 041 traits (Table S1). The UVMR analysis identified 189 traits with causal relationships (Table S2). Given the extensive number of lipid traits and their causal associations, we retained only those positive associations identified by the primary method, the IVW method, for further analysis. For duplicate exposures, we selected the most recent and largest GWAS datasets to ensure the robustness of the IVs and the precision of the association estimates, thereby enhancing statistical efficiency. Ultimately, we included 66 traits with causal associations in our analysis. The details of the data sources used, including trait names, GWAS IDs, authors or journals/consortia, sample sizes, populations, and PubMed IDs, are provided in Table S3. In clinical practice, basic lipid tests, including total cholesterol (TC), TG, HDL‐C, LDL‐C, as well as Apo A1, Apo B, and Lp(a), are commonly used, with increasing attention being given to the clinical significance of Apo A1, Apo B, and Lp(a). Therefore, we focused on these indicators to explore their potential causal associations with alopecia diseases and their clinical relevance.

### 3.1 Univariable Mendelian Randomization Analysis of Lipids and Hair Loss Diseases

**FIGURE 3:**
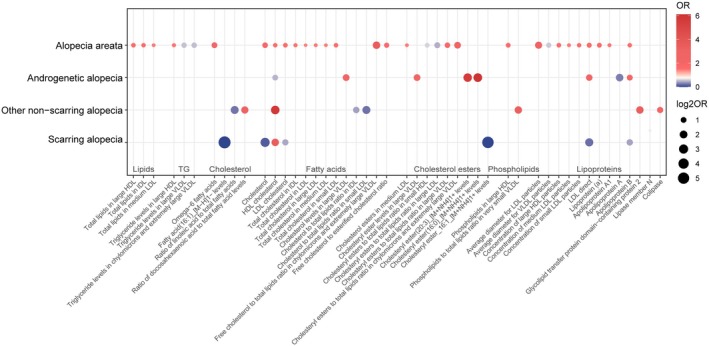
Bubble heatmap of lipid‐hair loss associations. This heatmap displays associations between various lipid classes and four hair loss conditions. Each bubble's size reflects the association's strength (|log2 OR|), with color intensity indicating the effect magnitude. OR, Odds ratio; TG, Triglycerides.

**FIGURE 4:**
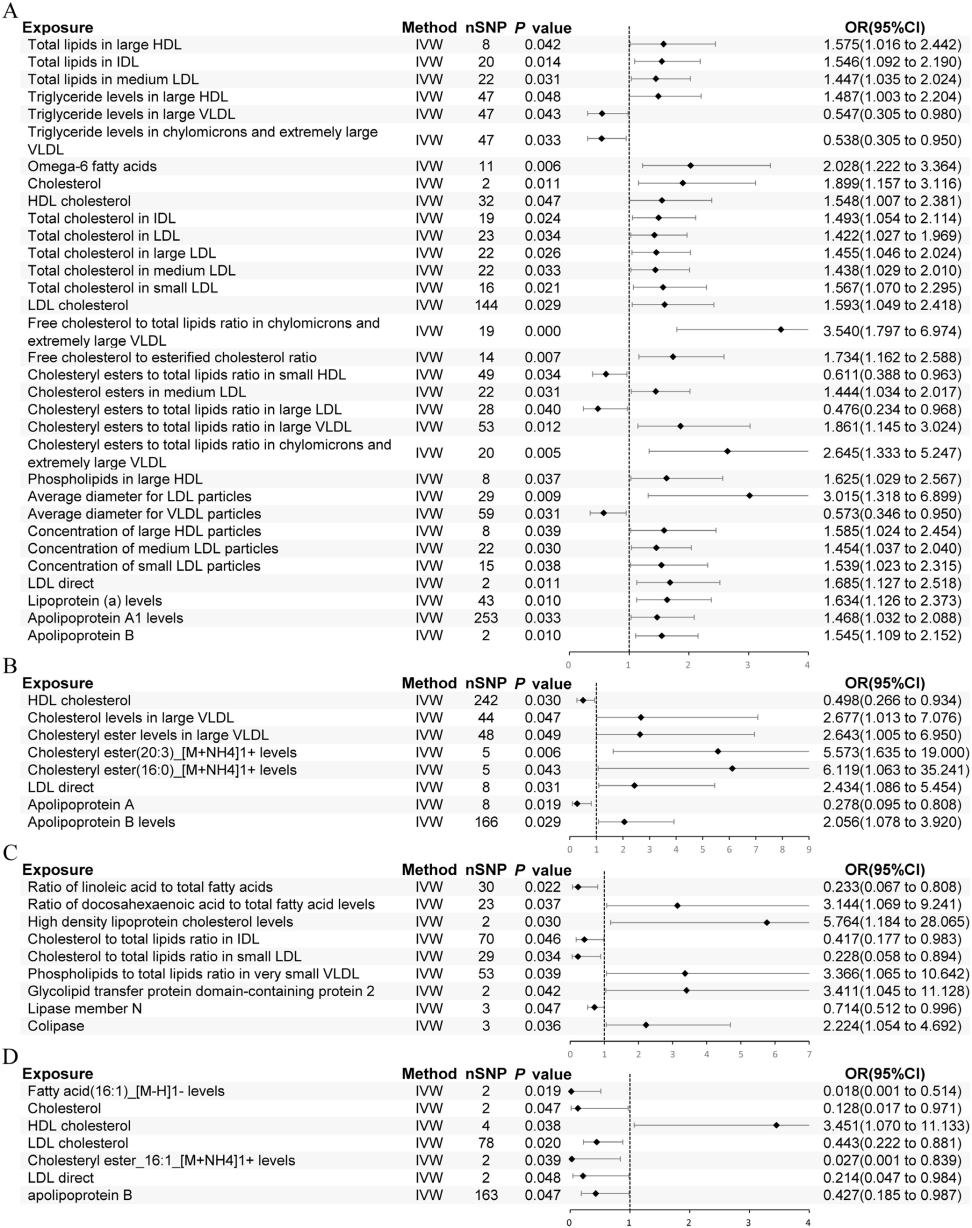
Univariable Mendelian randomization results for hair loss types. Results of the inverse‐variance weighted (IVW) method for (A) alopecia areata, (B) androgenetic alopecia, (C) non‐scarring hair loss, and (D) scarring hair loss are presented. OR, Odds ratio; CI, Confidence interval.

In AA, 41 lipid‐related traits with causal associations were identified, including 4 associated with lipids, 4 with TG, 1 with fatty acids, 13 with cholesterol, 6 with cholesterol esters, 3 with phospholipids, and 10 with lipoproteins (Figure 3). Notably, cholesterol (nSNP = 2, *p* = 0.011, OR [95% CI]: 1.899 [1.157 to 3.116]), HDL‐C (nSNP = 32, *p* = 0.047, OR [95% CI]: 1.548 [1.007 to 2.381]), LDL‐C (nSNP = 144, *p* = 0.029, OR [95% CI]: 1.593 [1.049 to 2.418]), Apo A1 (nSNP = 253, *p* = 0.033, OR [95% CI]: 1.468 [1.032 to 2.088]), Apo B (nSNP = 2, *p* = 0.010, OR [95% CI]: 1.545 [1.109 to 2.152]), and Lp(a) (nSNP = 43, *p* = 0.010, OR [95% CI]: 1.634 [1.126 to 2.373]) were significantly associated with AA (Figure 4A). Additionally, triglyceride levels in large very low‐density lipoprotein (VLDL), chylomicrons, and very large VLDL, as well as the ratio of cholesterol ester to total lipids in small HDL and large LDL and the average diameter of VLDL particles may act as protective factors (Figure 4A).

For AGA, 8 traits showed significant causal associations, including 2 related to cholesterol, 3 to cholesterol esters, and 3 to lipoproteins (Figure 3). HDL‐C (nSNP = 242, *p* = 0.030, OR [95% CI]: 0.498 [0.266 to 0.934]) and Apo B (nSNP = 166, *p* = 0.029, OR [95% CI]: 2.056 [1.078 to 3.920]) were identified as significant factors, and also Apo A was noted as a potential protective factor for AGA (Figure 4B).

In non‐scarring alopecia, 9 traits with causal associations were detected, including 2 related to fatty acids, 3 to cholesterol, 1 to phospholipids, and 3 to lipoproteins (Figure 3). High levels of HDL‐C (nSNP = 2, *p* = 0.030, OR [95% CI]: 5.764 [1.184 to 28.065]) were associated with an increased risk of this condition (Figure 4C). Protective factors may include the ratio of linoleic acid (LA) to total fatty acids, the ratio of cholesterol to total lipids in IDL and small LDL, and Lipase member N (Figure 4C).

In scarring alopecia, 8 traits with causal associations were observed, including 1 related to fatty acids, 3 to cholesterol, 1 to cholesterol esters, and 3 to lipoproteins (Figure 3). Significant associations were identified for cholesterol (nSNP = 2, *p* = 0.047, OR [95% CI]: 0.128 [0.017 to 0.971]), HDL‐C (nSNP = 4, *p* = 0.038, OR [95% CI]: 3.451 [1.070 to 11.133]), LDL‐C (nSNP = 78, *p* = 0.020, OR [95% CI]: 0.443 [0.222 to 0.881]), and Apo B (nSNP = 163, *p* = 0.047, OR [95% CI]: 0.427 [0.185 to 0.987]) (Figure 4D). Cholesterol‐lowering drugs were identified as protective against this condition (nSNP = 41, *p* = 0.030, OR [95% CI]: 0.008 [0.000 to 0.627]) (Table S2).

These causal relationships were consistent across other methods, such as the WM, MR‐Egger regression, and the weighted mode method, with similar causal estimates in direction and magnitude (Table S4). Scatter plots and funnel plots are shown in Figures S1–S4.

Cochran's Q test did not reveal significant heterogeneity (*p* > 0.05) (Table S5–S8). However, some lipid traits, including total lipids in large LDL, the ratio of TG to total lipids in chylomicrons and very large VLDL, total cholesterol levels, serum TC, the ratio of cholesterol to total lipids in chylomicrons and extremely large VLDL, cholesterol esters in large VLDL, phospholipids in IDL and medium LDL, and large LDL particles concentration, were associated with AA, while small HDL particles concentration was linked to scarring alopecia, showing pleiotropy (*p* < 0.05); the remaining 56 causal relationships did not show signs of horizontal pleiotropy (*p* > 0.05) (Tables S5–S8). Figure 4 presents the forest plots of exposure and outcomes after sensitivity analysis. The leave‐one‐out test indicated that the observed associations were not driven by any single SNP, suggesting that the results are reliable and stable (Figure S5).

### 3.2 Multivariable Mendelian Randomization Analysis of Lipids and Hair Loss Diseases

Using MVMR analysis, we investigated the consistent and direct causal effects of specific exposures on outcomes while controlling for other exposures. Significant risk factors from the univariable analysis, including HDL‐C, LDL‐C, and TG, were included, while Apo A1 and Apo B were excluded to avoid multicollinearity issues because Apo A1 is the main structural and functional protein of HDL‐C, whereas Apo B encapsulates LDL‐C and TG to form particles [30, 31, 32].

**FIGURE 5:**
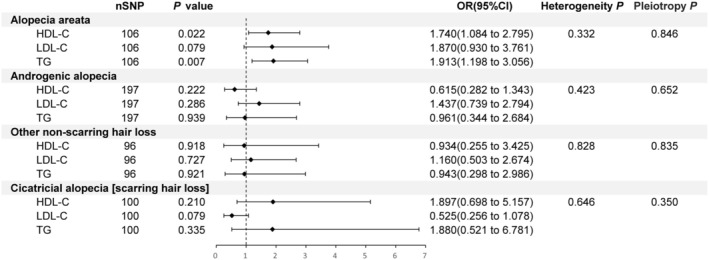
Multivariable Mendelian randomization (MVMR) results using the inverse‐variance weighted (IVW) method. This figure shows the MVMR findings for hair loss diseases using the IVW approach, along with *p* values for pleiotropy and heterogeneity tests, indicating the robustness of the results.

For AA, 106 IVs were selected for MVMR analysis, revealing a positive association between higher levels of HDL‐C and TG with the risk of developing the condition (*p* = 0.022, OR [95% CI]: 1.740 [1.084 to 2.795]; *p* = 0.007, OR [95% CI]: 1.913 [1.198 to 3.056]), while LDL‐C was not significantly associated with AA (*p* > 0.05) (Figure 5). This finding contrasts with the UVMR analysis, where HDL‐C and LDL‐C were positively correlated with AA, but TG showed no causal relationship. For the other three outcomes, no significant causal relationships were found after adjusting for other exposures (*p* > 0.05) (Figure 5). These relationships had similar causal effect estimates and directions across other methods (Table S9). Cochran's Q test indicated no heterogeneity, and the MR‐PRESSO test and MR‐Egger intercept method did not show horizontal pleiotropy (Figure 5, Table S10).

## 4 Discussion

In this large‐scale, comprehensive MR study, supported by extensive GWAS data, we employed both UVMR and MVMR to explore the causal relationships between 983 lipid traits and hair loss diseases. Our findings revealed 189 associations, of which 66 were statistically significant, with 56 being both significant and robust. The consistency of IVW results across various MR methods, coupled with the absence of outliers in MR‐PRESSO estimates, along with tests for heterogeneity and horizontal pleiotropy, underscores the robustness of our conclusions. These findings provide substantial evidence of the potential causal effects of various lipid traits on four types of hair loss diseases.

Regarding AA, multiple lipid categories—including TG, fatty acids, cholesterol, cholesteryl esters, phospholipids, and lipoproteins—demonstrated statistically significant causal relationships. Elevated HDL‐C, LDL‐C, Apo A1, Apo B, and Lp(a) were positively associated with disease progression. Notably, a positive correlation between Omega‐6 fatty acids and the risk of AA was observed. Given that potential interactions between lipids may lead to unstable causality, we adjusted for lipid composition using MVMR. The results showed that the causal relationship between HDL‐C and AA remained robust, suggesting that high HDL‐C may be an independent risk factor for AA. However, the causal association between LDL‐C and AA dissipated, and TG showed a significant causal relationship. This difference likely arises from multicollinearity among HDL‐C, LDL‐C, and TG. Whereas independent analyses may obscure the independent effects of one exposure due to the strong correlations among these variables, MVMR, by considering the correlations among exposure variables and adjusting for their mutual effects, controls potential confounders and reveals the independent effects of specific exposures, improving the robustness of the results [33].

AA is an autoimmune inflammatory disease characterized by non‐scarring hair loss due to immune responses targeting hair follicles. Recent research suggests that abnormal lipid levels may play a significant role in AA pathogenesis. A retrospective case–control study evaluating monocyte HDL‐C ratio (MHR) in the blood of 70 patients with AA and 70 healthy controls found a statistically significant increase, suggesting it could be used as a diagnostic marker for AA [34]. Additionally, a clinical analysis by Chean et al. involving blood lipid tests in 752 AA patients and 21 538 controls found a significant increase in LDL‐C among female AA patients [14]. Conversely, Anna et al.'s study of lipid levels in 52 AA patients found no significant differences in HDL‐C, LDL‐C, and TG levels compared to healthy controls [35]. These discrepancies may be due to variations in sample size, participant characteristics, or measurement methods. However, the specific role of lipids in AA remains unclear. Serum lipids are crucial for lipid metabolism in the dermis and the hair follicle microenvironment. Adipocytes and their precursor cells are actively involved in the hair growth cycle [36, 37] and influence pathological changes in AA patients through lipid peroxidation reactions [38, 39, 40]. Inflammation also plays a significant role in AA. For instance, Mineo's study found that HDL‐C in endothelial cells and leukocytes exhibits anti‐inflammatory properties, stimulates the production of adipocyte‐derived cytokine adiponectin, and promotes insulin secretion [41], which has been validated in several case–control studies [35, 42]. Similarly, Omega‐6 fatty acid metabolites, such as arachidonic acid, can be converted into inflammatory lipid mediators like leukotriene B4 (LTB4) and prostaglandin E2 (PGE2), which may promote inflammation, inhibit hair growth, and cause follicular damage [43, 44, 45]. What is more, Lp(a), an LDL‐like particle bound to Apo A, has been associated with various diseases, including cardiovascular diseases [46]. Although the impact of Lp(a) on hair loss remains unclear, it is hypothesized to affect hair follicles by promoting the release of inflammatory mediators, leading to inflammation around the follicles and disrupting their normal function and growth cycle. Additionally, research by Hironobu et al. confirmed that obesity induced by a high‐fat diet can lead to lipid droplet accumulation in hair follicle stem cells and activate NF‐κB, suppressing the Sonic Hedgehog (Shh) signaling pathway, leading to hair follicle stem cell depletion, follicle miniaturization, and ultimately hair loss [47]. Therefore, the mechanisms by which lipids contribute to AA likely involve multiple aspects, including effects on dermal lipid metabolism and the hair follicle microenvironment, regulation of inflammatory responses, secretion of adipokines and insulin, and signaling.

As for AGA, UVMR analysis revealed that high HDL‐C is a protective factor, whereas high Apo B is a risk factor for AGA. A positive correlation between cholesteryl esters and disease progression was also observed. However, after controlling for LDL‐C and TG in MVMR, the causal association between HDL‐C and AGA was no longer statistically significant, suggesting that LDL‐C and TG may influence the effect of HDL‐C. When these variables are controlled, the independent effect of HDL‐C may be diminished.

AGA is characterized by a specific pattern of hair loss on the scalp, with genetic factors and androgens being key contributors. Multiple observational studies have supported the potential role of lipid metabolism abnormalities in AGA. A meta‐analys, is revealed that serum TC, LDL‐C, and TG levels were significantly higher in AGA patients compared to controls, while HDL‐C levels were significantly lower [16]. A cross‐sectional survey involving 740 participants found a strong association between AGA and HDL‐C levels [48]. Several case–control studies analyzing blood lipid parameters in AGA patients also found significantly higher levels of Lp(a) in the study group compared to controls. Recent advancements in understanding AGA pathogenesis have revealed that its pathological process mainly involves gradual miniaturization of hair follicles and changes in hair cycle dynamics [45]. Cholesterol is pivotal in the pathogenesis of AA, functioning as a key component of the epidermal permeability barrier, steroid hormone synthesis, and the proliferation and differentiation of keratinocytes [17]. Secondly, downregulation of Wnt/βcatenin signaling is a critical mechanism in AGA, with signaling facilitated by cholesterol and cholesterol‐rich lipid rafts [49]. High levels of the cholesterol metabolite DHT [50, 51] increase androgen receptor activity while inhibiting Wnt/β‐catenin signaling [52], accelerating follicular degeneration. Additionally, cholesterol released by dermal adipocytes can be taken up by follicular cells, potentially influencing follicular steroidogenesis and the transition from early to mid‐anagen phases [53]. Research by Zhang et al. demonstrated that mutations in the mouse glycerol kinase 5 (GK5) gene result in the toku phenotype, characterized by progressive hair loss and increased free cholesterol and cholesterol ester levels, further supporting the crucial role of cholesterol balance in normal follicular function [54]. Thus, lipids may play a significant role in AGA through various mechanisms, including promoting follicle miniaturization, regulating Wnt/β‐catenin signaling, enhancing androgen receptor activity, and influencing follicular steroidogenesis and hair growth phase transitions via cholesterol released from dermal adipocytes.

UVMR analysis indicated that HDL‐C is a risk factor for non‐scarring alopecia, whereas MVMR analysis showed no causal relationship. A high proportion of LA in total fatty acids was negatively correlated with the progression of non‐scarring alopecia, whereas a high proportion of docosahexaenoic acid (DHA) in total fatty acids was positively correlated.

Non‐scarring alopecia includes a diverse group of disorders characterized by reversible hair loss, primarily resulting from disruptions in the hair cycle while preserving hair follicles. Unlike AA, which is immune‐mediated, and AGA, which is driven by hormonal factors, non‐scarring alopecia arises from varied etiologies, including inflammation, psychological stress, and metabolic disturbances. LA and DHA are essential polyunsaturated fatty acids. LA, the predominant 18‐carbon n‐6 polyunsaturated fatty acid in normal epidermis, is found mainly in various plant oils, nuts, and seeds. LA and its metabolites have anti‐inflammatory and anti‐proliferative effects, and LA deficiency can lead to specific scaly skin disorders and excessive transepidermal water loss [55]. Studies have shown that LA treatment of human hair dermal papilla cells can activate Wnt/β‐catenin signaling and promote cell growth by increasing the expression of cyclins such as Cyclin D1 and CDK2, thereby alleviating hair loss [18]. However, excessive LA intake can lead to the production of oxidized linoleic acid metabolites (OXLAM), causing chronic diseases like obesity, cardiovascular diseases, and cancer [56], which may negatively affect hair growth. Naito's study demonstrated that the topical application of LA hydroperoxide (a type of lipid peroxide) accelerates the onset of the catagen phase in the hair cycle of mice. This effect is likely due to lipid peroxides inducing apoptosis in hair follicle cells and promoting apoptosis in human epidermal keratinocytes by upregulating apoptosis‐related genes [57]. DHA, an omega‐3 fatty acid abundant in the brain and retina, is available from sources such as fish oil and breast milk. DHA‐derived resolvins and protectins exhibit anti‐inflammatory properties, suggesting a novel mechanism by which n‐3 polyunsaturated fatty acids modulate inflammatory processes [58]. Hao et al. found that mice on a high‐fat diet enriched with fish oil experienced hair loss, potentially due to atypical infiltration of CD207‐ (langerin‐) bone marrow‐derived macrophages in the dermis, which exacerbated hair loss through enhanced TNF‐α signaling. Mechanistically, epidermal fatty acid‐binding protein (E‐FABP) has been implicated in TNF‐α‐mediated hair loss in dermal macrophages by activating the n‐3 fatty acid/ROS/IL‐36 signaling pathway [59]. Conversely, Takahata et al. suggested that DHA might protect against chemotherapy‐induced alopecia by modulating apoptosis [60].

Cicatricial alopecia, characterized by follicular damage, scarring of the skin, and permanent hair loss. HDL‐C was identified as a risk factor for cicatricial alopecia, while LDL‐C and Apo B appeared to be protective factors, though these associations were not confirmed in multivariate analyses. Additionally, fatty acids showed an inverse correlation with the risk of cicatricial alopecia. Cholesterol has an important role in its pathogenesis [53, 54].

Abnormal lipid levels, particularly elevated TG and HDL‐C, are key MetS features, alongside other indicators such as blood glucose, blood pressure, and abdominal fat [61]. Multiple systematic reviews and meta‐analyses have indicated that AA may be an independent risk factor associated with MetS [62, 63, 64]. Similarly, research by F. Acibucu et al. found that patients with early‐onset AGA exhibited a significantly higher prevalence of insulin resistance (IR), hyperinsulinemia, and MetS, all of which are independent risk factors for coronary artery disease (CAD) [65]. Given these findings, it is crucial to manage lipid levels in patients with hair loss. Dietary interventions, such as the Mediterranean diet, have significantly improved hair loss due to its low saturated fat content and high levels of unsaturated fats, polyphenols, and antioxidants [66]. Additionally, supplements containing omega‐3, omega‐6, and antioxidants have significantly improved hair loss progression [67]. Notably, studies on atherosclerotic mouse models (Apo E−/−) revealed that long‐term consumption of a high‐fat, high‐cholesterol diet leads to hair discoloration and loss [68]. Regarding pharmacological treatment, cholesterol‐lowering drugs such as simvastatin and ezetimibe can be effective in treating AA, which is consistent with our study's findings. Although the precise mechanisms remain unclear, the major histocompatibility complex II (MHC‐II) and intercellular adhesion molecule‐1 (ICAM‐1) are believed to play critical roles in the immunomodulatory effects of statins and the pathophysiology of autoimmune hair loss [69, 70]. Since MetS can lead to cardiovascular disease and type 2 diabetes, addressing hair loss in these patients may have positive implications for their cardiovascular and metabolic health [71].

This study is the first to utilize dual‐sample UVMR and MVMR methods to elucidate the causal relationship between lipid levels and hair loss disorders. With an unprecedentedly large sample size, the study systematically explores potential risk factors for hair loss. Previous studies on lipid levels and hair loss typically measured lipid levels only once. In contrast, MR methods enabled more precise causal inferences at the genetic level, helping to overcome the limitations of traditional observational and cross‐sectional studies, where confounding factors such as metabolic disorders and reverse causality can undermine reliability. Furthermore, through MVMR analysis, we not only clarified the independent effects of each the lipid variable but also examined their interactions, thereby avoiding the biases that might arise from univariable analysis. In terms of results, we employed various computational methods and statistical tools, and the estimates from different MR methods showed consistent trends. The MR‐PRESSO method was used to identify potential outliers, and sensitivity analyses indicated no heterogeneity or horizontal pleiotropy. Leave‐one‐out analysis, which detects associations driven by a single IV, revealed no anomalies. These results emphasize the robustness of our findings and support the validity of our causal inferences. By identifying specific lipid metabolic pathways associated with different types of hair loss disorders, our study provides insights into potential biological mechanisms underlying alopecia. Further research is needed to evaluate whether therapies targeting lipid metabolism could potentially emerge as novel strategies for treating hair loss.

However, this study has certain limitations. Firstly, the reliance on summary‐level GWAS data, rather than individual‐level data, restricts the ability to perform sensitivity analyses at the individual level, such as stratification based on demographic or clinical characteristics, adjusting for potential confounders, or conducting subgroup analyses. Secondly, despite the large overall sample size and the broad and accurate selection of exposures, the definitions of non‐scarring and scarring hair loss outcomes are general. Non‐scarring hair loss encompasses a variety of conditions, including, but not limited to, telogen effluvium (TE), anagen effluvium, and trichotillomania. Scarring hair loss, on the other hand, includes but is not limited to, lymphocyte‐associated lichen planopilaris (LPP), neutrophil‐associated folliculitis decalvans, and mixed inflammatory infiltrate erosive pustular dermatitis of the scalp. Due to the lack of GWAS data for these phenotypes, we were unable to assess the causal relationships between lipids and different clinical phenotypes of hair loss diseases, so the causal relationships obtained may be mixed or inaccurate. Therefore, caution should be exercised when interpreting the causality estimates for these categories, considering the potential for heterogeneity. Additionally, as the diagnosis of hair loss diseases in FinnGen is based on ICD codes without dermatological validation, potential misclassification may exist. Future studies incorporating clinically confirmed cases and more granular alopecia subtypes would help refine these associations. Thirdly, although MR mitigates confounding and reverse causality, it has inherent limitations. Some lipid traits were instrumented by fewer than 10 SNPs. Although we ensured that the F‐statistic (F > 10) met the threshold to mitigate weak instrument bias, its potential influence on the reliability of our causal estimates cannot be completely excluded. Additionally, pleiotropy remains a key concern, as genetic variants may affect hair loss through pathways unrelated to lipid levels. To address this, we applied MR‐PRESSO to detect and correct for horizontal pleiotropy and the MR‐Egger intercept test to assess directional pleiotropy, ensuring the robustness of our findings. Furthermore, we accounted for potential interactions between lipid traits using MVMR. However, residual pleiotropy cannot be entirely ruled out. Finally, most of the cohorts in this study were derived from European populations, and further research and validation are needed to determine whether these results can be generalized to other ethnic populations. Moreover, although our study explored the potential mechanisms behind the causal relationship between lipids and hair loss diseases, further mechanistic studies and clinical trials are needed for validation.

## 5 Conclusion

This study utilized dual‐sample UVMR and MVMR methods to investigate the potential causal relationships between lipid‐related traits and hair loss disorders. The UVMR analysis demonstrated that lipid levels significantly influence the risk of hair loss disorders. The MVMR analysis further revealed independent causal effects of HDL‐C, LDL‐C, and TG on these diseases. By examining the metabolic risk factors of hair loss from a genetic perspective, this study highlights the crucial role of lipids in the pathogenesis of hair loss disorders, offering new theoretical foundations and intervention targets for the prevention and treatment of these diseases.

## Author Contributions

Y.X., R.B., and L.R. contributed to the conceptualization, methodology, investigation, analysis, visualization, and writing – review and editing. Y.X. and R.B. were responsible for methodology and data curation. L.R. and H.F. contributed to visualization, validation, and writing – review and editing. H.T. and L.D. were involved in investigation, writing – review, and editing. C.F., X.Z., and Z.L. handled data curation and writing – review. All authors contributed to the planning, execution, and analysis of the study, and they reviewed and approved the final submitted version.

## Ethics Statement

All analyses were conducted using publicly available data from the IEU Open GWAS Project (https://gwas.mrcieu.ac.uk/). As such, no additional ethical approvals were required.

## Conflicts of Interest

The authors declare no conflicts of interest.

## Supporting information


**Figures S1–S2.** Scatter plots depicting the effect of exposure on outcomes using different methods.
**Figures S3–S4.** Funnel plots showing the effect of exposure on outcomes using different methods.
**Figure S5.** Leave‐One‐Out analysis results for the trait’s effect on various types of hair loss disorders.

**Table S1.** Summary of GWAS information for lipid‐related traits included in the study (*N* = 983).
**Table S2.** Univariable mendelian randomization analysis results (*N* = 189).
**Table S3.** Summary of GWAS information on exposures and outcomes in the study.
**Table S4.** Univariable mendelian randomization analysis results for IVW positivity (*N* = 66).
**Table S5.** Sensitivity analysis results for alopecia areata.
**Table S6.** Sensitivity analysis results for androgenic alopecia.
**Table S7.** Sensitivity analysis results for other non‐scarring hair loss.
**Table S8.** Sensitivity analysis results for cicatricial alopecia [scarring hair loss].
**Table S9.** Multivariable Mendelian randomization estimates for HDL‐C, LDL‐C, and TG.
**Table S10.** Multivariable MR‐PRESSO results for HDL‐C, LDL‐C, and TG.

## Data Availability

The datasets used in this study are publicly accessible from the IEU Open GWAS Project (http://gwas.mrcieu.ac.uk/). Further information can be found in the article or Supporting Information. For additional inquiries, please contact the corresponding authors.
