# Supplementary material for: Potential Causal Relationship Between Extensive Lipid Profiles and Various Hair Loss Diseases: Evidence From Univariable and Multivariable Mendelian Randomization Analyses
Source: J Cosmet Dermatol. 2025 Apr 10;24(4):e70176. doi: 10.1111/jocd.70176 (PMC11984456; doi:10.1111/jocd.70176)
Supplement: Supplementary file 1 — Figures S1–S2. Scatter plots depicting the effect of exposure on outcomes using different methods. Figures S3–S4. Funnel plots showing the effect of exposure on outcomes using different methods. Figure S5. Leave‐One‐Out analysis results for the trait’s effect on various types of hair loss disorders. [file JOCD-24-e70176-s002.docx]

**Supplementary Materials**

Potential Causal Relationship between Extensive Lipid Profiles and Various Hair Loss Diseases: Evidence from Univariable and Multivariable Mendelian Randomization Analyses

**Supplemental Information**

**Supplementary Figure 1-2.** Scatter plots depicting the effect of exposure on outcomes using different methods.

**Supplementary Figure 3-4.** Funnel plots showing the effect of exposure on outcomes using different methods.

**Supplementary Figure 5.** Leave-One-Out analysis results for the trait's effect on various types of hair loss disorders.

**Supplementary Table 1** Summary of GWAS information for lipid-related traits included in the study (N = 983).

**Supplementary Table 2** Univariable mendelian randomization analysis results (N=189).

**Supplementary Table 3** Summary of GWAS information on exposures and outcomes in the study.

**Supplementary Table 4** Univariable mendelian randomization analysis results for IVW positivity (N=66).

**Supplementary Table 5** Sensitivity analysis results for alopecia areata.

**Supplementary Table 6** Sensitivity analysis results for androgenic alopecia.

**Supplementary Table 7** Sensitivity analysis results for other nonscarring hair loss.

**Supplementary Table 8** Sensitivity analysis results for cicatricial alopecia [scarring hair loss].

**Supplementary Table 9** Multivariable mendelian randomization estimates for HDL-C, LDL-C and TG.

**Supplementary Table 10** Multivariable MR-PRESSO results for HDL-C, LDL-C and TG.

**Supplementary Figures**


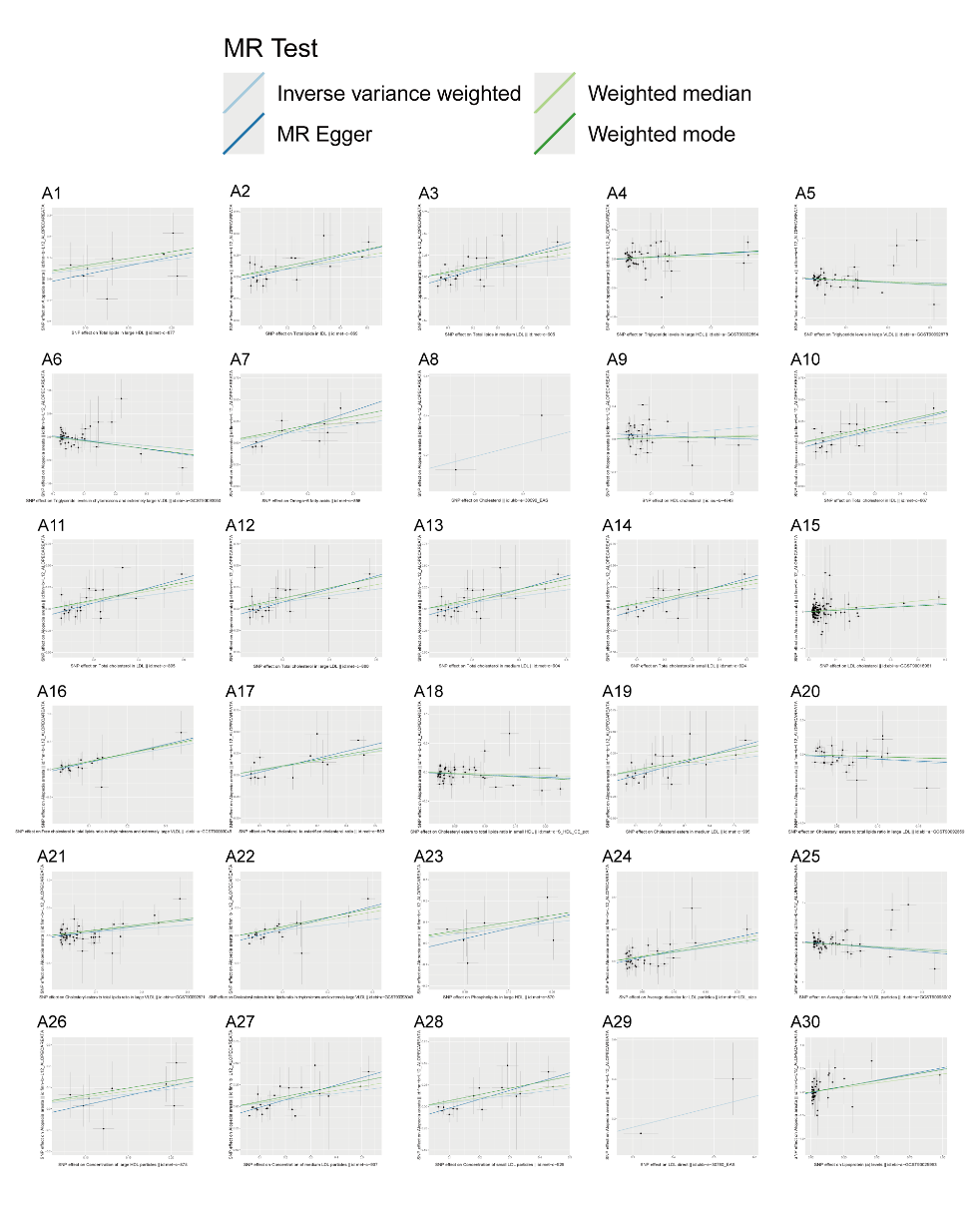

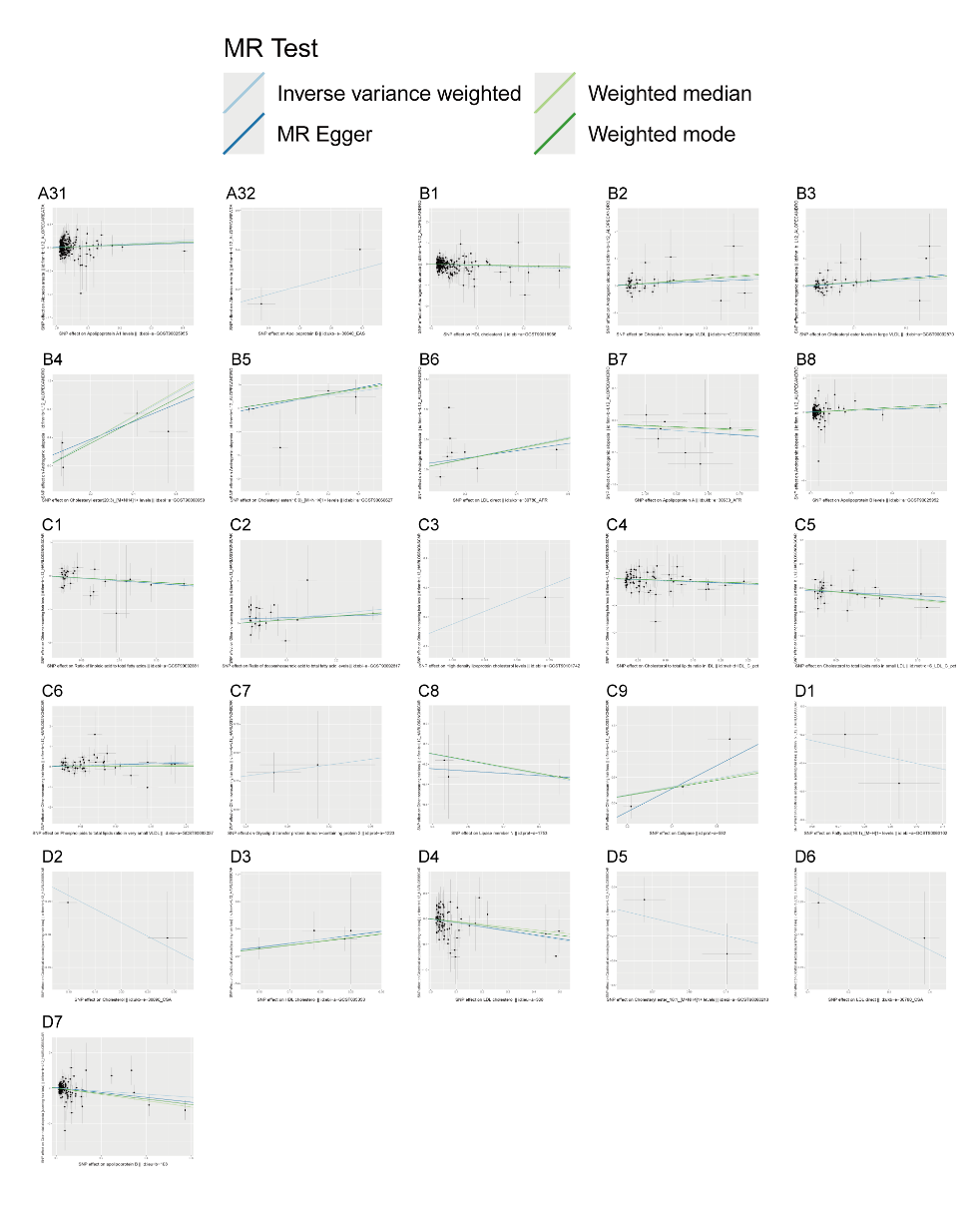


**Supplementary Figure 1-2. Scatter plots depicting the effect of exposure on outcomes using different methods.** Panels A, B, C, and D represent the effect of the trait on alopecia areata, androgenetic alopecia, other non-scarring hair loss, and scarring hair loss, respectively.


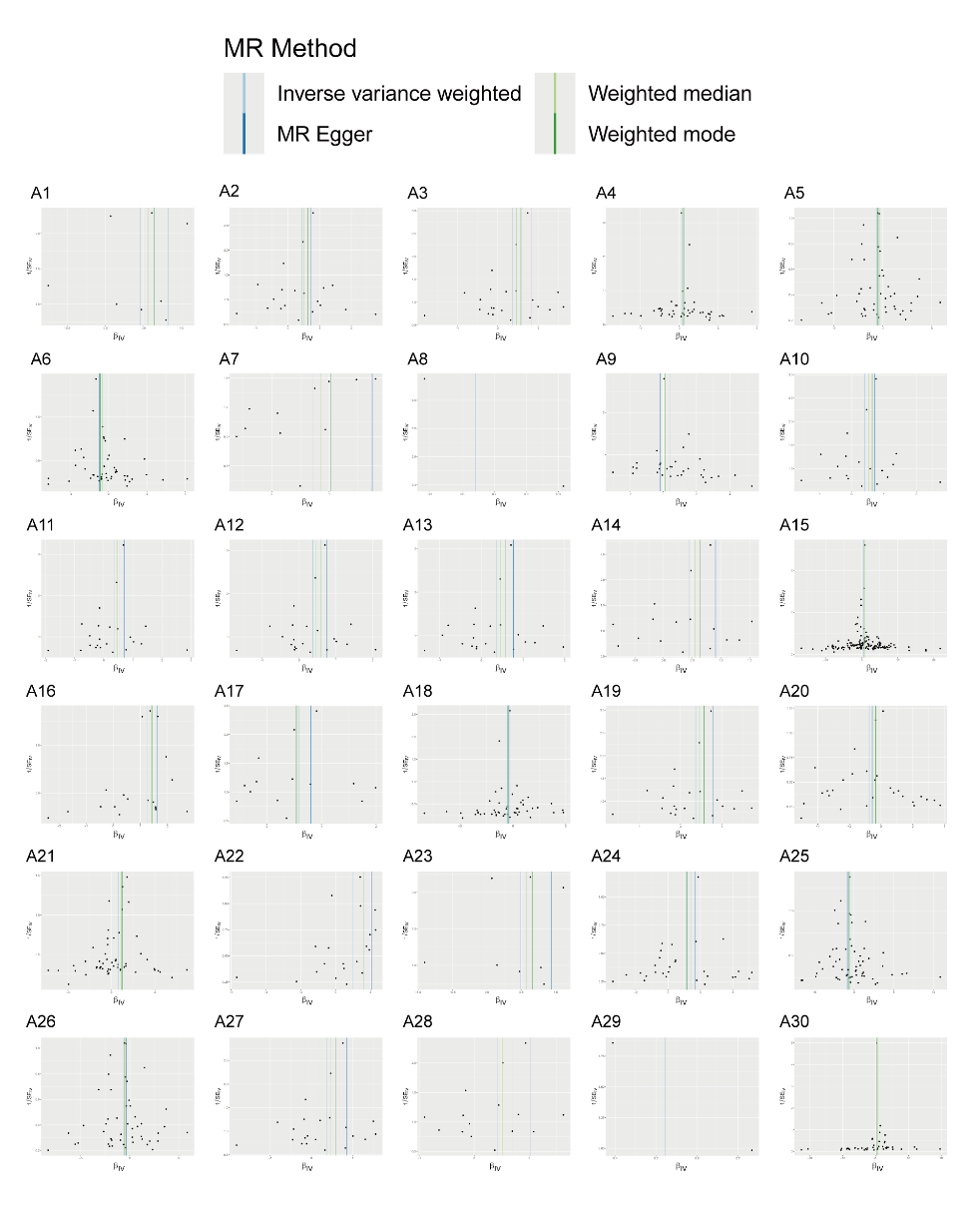

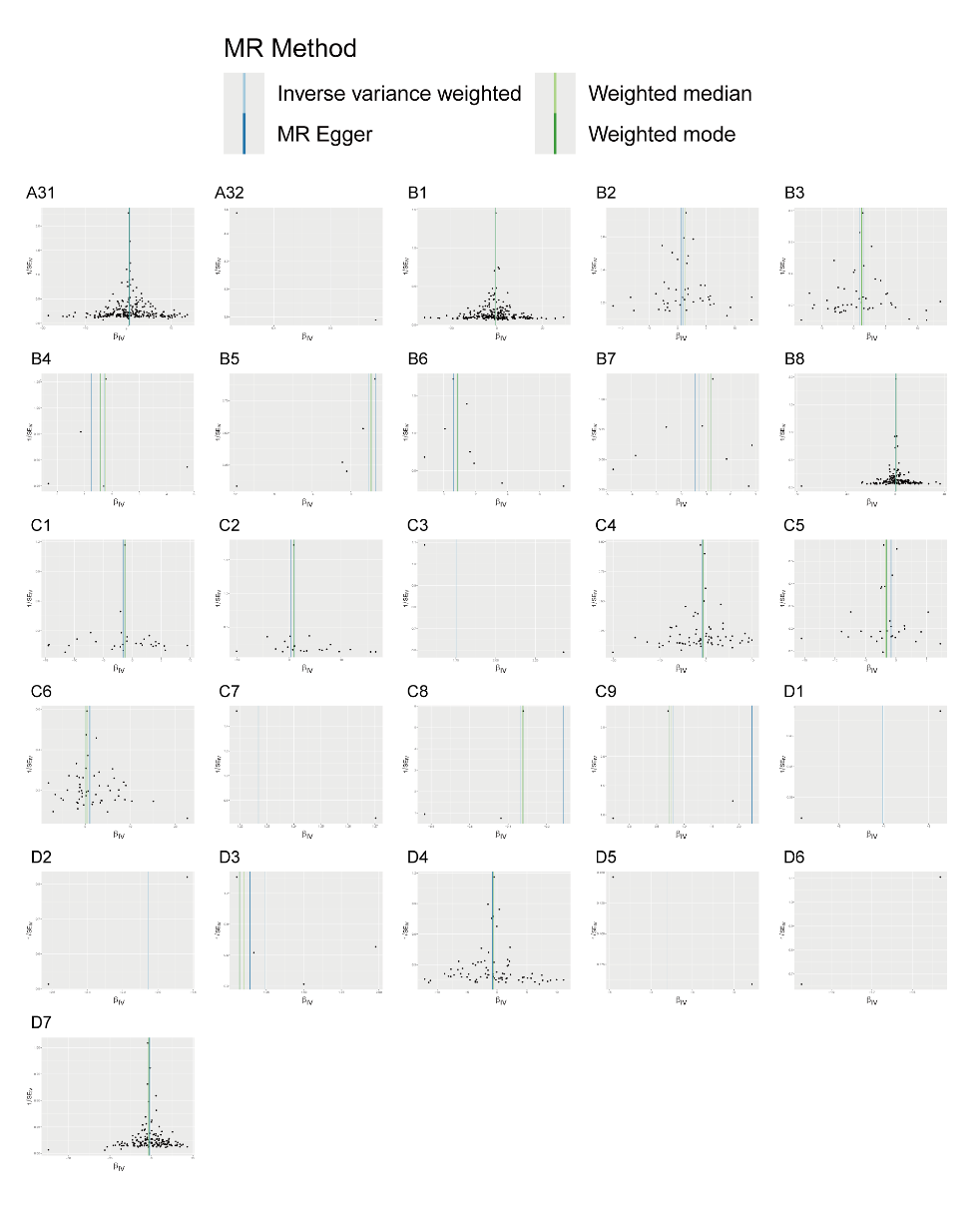
**Supplementary Figure 3-4. Funnel plots showing the effect of exposure on outcomes using different methods.** Panels A, B, C, and D represent the effect of the trait on alopecia areata, androgenetic alopecia, other non-scarring hair loss, and scarring hair loss, respectively. The x-axis represents the SNP-exposure effect, while the y-axis represents the inverse of the standard error of the SNP-outcome effect.


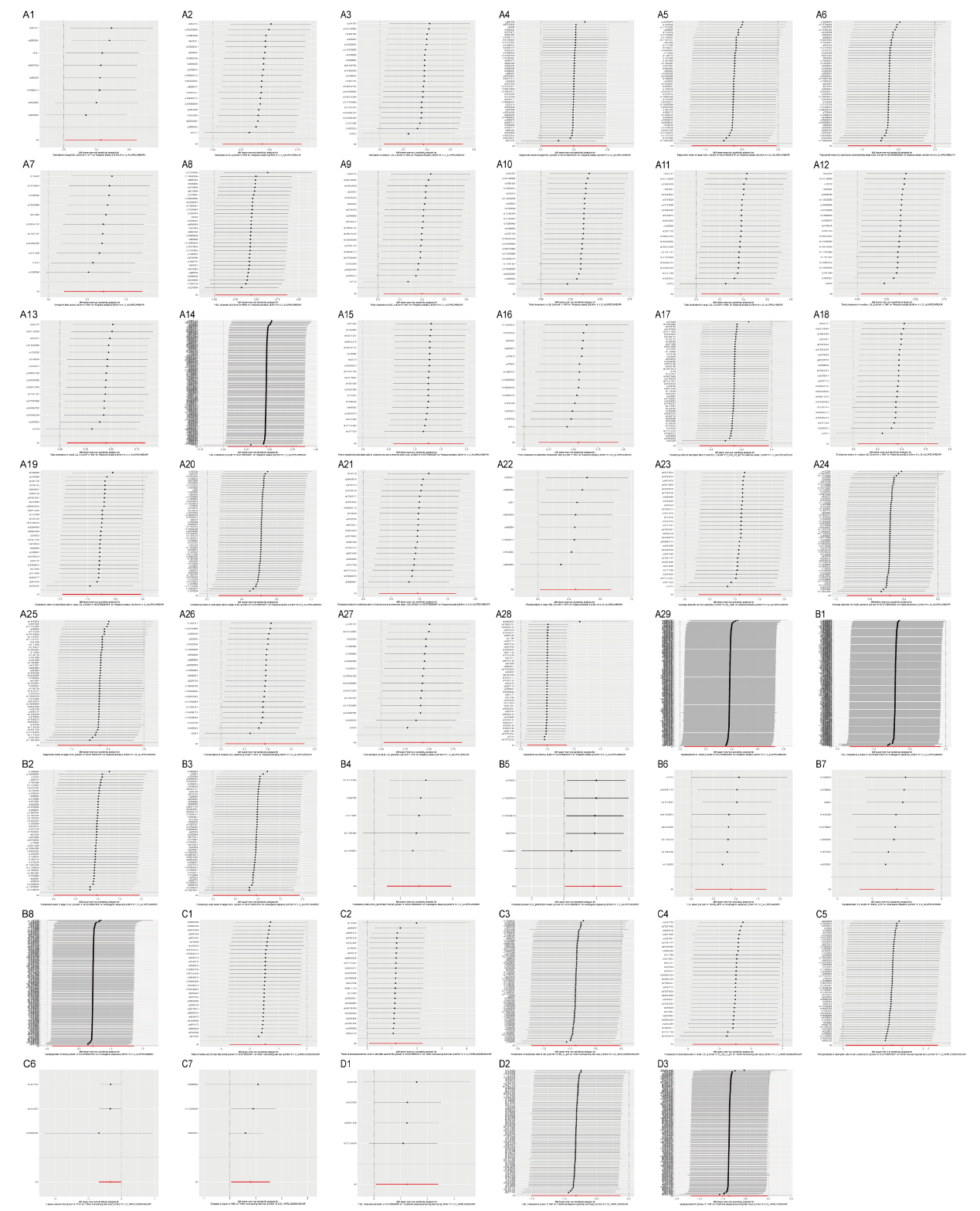


**Supplementary Figure 5. Leave-One-Out analysis results for the trait's effect on various types of hair loss disorders.** Panels A, B, C, and D represent the effect of the trait on alopecia areata, androgenetic alopecia, other non-scarring hair loss, and scarring hair loss, respectively.
